# Thrombus leukocytes exhibit more endothelial cell-specific angiogenic markers than peripheral blood leukocytes do in acute coronary syndrome patients, suggesting a possibility of trans-differentiation: a comprehensive database mining study

**DOI:** 10.1186/s13045-017-0440-0

**Published:** 2017-03-23

**Authors:** Hangfei Fu, Nish Vadalia, Eric R. Xue, Candice Johnson, Luqiao Wang, William Y. Yang, Claudette Sanchez, Jun Nelson, Qian Chen, Eric T. Choi, Jian-Xing Ma, Jun Yu, Hong Wang, Xiaofeng Yang

**Affiliations:** 1 Department of Pharmacology, Lewis Katz School of Medicine at Temple University, Philadelphia, PA 19140 USA; 2 Center for Metabolic Disease Research, Lewis Katz School of Medicine at Temple University, 3500 North Broad Street, MERB-1059, Philadelphia, PA 19140 USA; 3 Center for Cardiovascular Research, Lewis Katz School of Medicine at Temple University, 3500 North Broad Street, 10th Floor, Philadelphia, PA 19140 USA; 4 Center for Thrombosis Research, Lewis Katz School of Medicine at Temple University, Philadelphia, PA 19140 USA; 5 Department of Surgery, Lewis Katz School of Medicine at Temple University, Philadelphia, PA 19140 USA; 6 Department of Physiology, University of Oklahoma College of Medicine, Oklahoma City, OK 73104 USA; 7 Department of Cardiovascular Medicine, The Second Affiliated Hospital at Nanchang University, Nanchang, Jiangxi 330006 China

**Keywords:** Angiogenic genes, Tissue expression of genes, Pathological modulation of angiogenesis, Immune regulation of angiogenesis, Angiogenic leukocytes

## Abstract

**Background:**

Current angiogenic therapies for cancers and cardiovascular diseases have not yet achieved expected benefits, which reflects the need for improved understanding of angiogenesis. In this study, we focused on solving the problem of whether tissues have different angiogenic potentials (APs) in physiological conditions and how angiogenesis is regulated in various disease conditions.

**Methods:**

In healthy and diseased human and mouse tissues, we profiled the expression of 163 angiogenic genes, including transcription regulators (TRs), growth factors and receptors (GF/Rs), cytokines and chemokines (C/Cs), and proteases and inhibitors (P/Is). TRs were categorized as inflammatory, homeostatic, and endothelial cell-specific TRs, and C/Cs were categorized as pro-angiogenic, anti-angiogenic, and bi-functional C/Cs.

**Results:**

We made the following findings: (1) the human heart, muscle, eye, pancreas, and lymph node are among the tissues with the highest APs; (2) tissues with high APs have more active angiogenic pathways and angiogenic C/C responses; (3) inflammatory TRs dominate regulation of all angiogenic C/Cs; homeostatic TRs regulate all to a lower extent, while endothelial cell-specific TRs mainly regulate pro-angiogenic and bi-functional C/Cs; (4) tissue AP is positively correlated with the expression of oxygen sensors PHD2 and HIF1B, VEGF pathway gene VEGFB, and stem cell gene SOX2; (5) cancers of the digestive system tend to have increased angiogenesis dominated by endothelial cell-specific pro-angiogenic pathways, while lung cancer and prostate cancer have significantly decreased angiogenesis; and (6) endothelial cell-specific pro-angiogenic pathways are significantly increased in thrombus-derived leukocytes in patients with acute coronary artery disease.

**Conclusions:**

Our results demonstrate that thrombus-derived leukocytes express more endothelial cell-specific angiogenic markers to directly promote angiogenesis after myocardial infarction and that certain solid tumors may be more sensitive to anti-angiogenic therapies than others.

**Electronic supplementary material:**

The online version of this article (doi:10.1186/s13045-017-0440-0) contains supplementary material, which is available to authorized users.

## Background

Atherosclerosis and its complications, such as myocardial infarction, stroke, and peripheral artery disease (PAD), are the leading cause of morbidity and mortality in the world [1]. Tissue damage induced by ischemia and major artery blockage is one of the major pathological mechanisms underlying these diseases. Thus, development of novel therapeutics for re-gaining efficient blood supply of oxygen and nutrients by regenerating and remodeling vascular system in the ischemic tissues hold great promise for treating these life-threatening diseases [2]. In post-ischemic re-vascularization, the vascular system develops via the coordinated actions of several cell types for vasculogenesis [3], angiogenesis [4], arteriogenesis, and collateral growth [2]. In this study, we focus on angiogenesis, which is the process of sprouting new vessels from pre-existing ones [4]. Physiological angiogenesis occurs during development stages and wound healing, which consists of vessel destabilization, endothelial cell (EC) migration and proliferation, and sprouting. This is followed by the resolution phase, which is characterized by reduced EC proliferation and stabilization of the new vessel [4].

In ischemic diseases, inflammatory factors and cells play an important role in the mobilization of angiogenic cells [5], and the angiogenic process cross-talks with the inflammation process in many different ways [6]. To determine whether inflammatory factors assist angiogenic cells in recognizing cardiovascular disease risk factors such as hyperlipidemia, hyperglycemia, obesity, metabolic syndrome, hypertension, smoke, and hyperhomocystemia [7], we recently reported a series of findings on the expression and roles of innate immune/inflammation sensor caspase-1/inflammasome [8–10] in regulating angiogenic cells, including endothelial cell [11–15], Sca-1^+^ progenitor cell [16, 17]. We also found that CD4^+^Foxp3^+^ regulatory T cells (Tregs) inhibit vascular inflammation [18–20] and that Treg-generated newly classified responsive immunosuppressive cytokine interleukin-35 (IL-35) [21] suppresses EC activation [22]. Also, several angiogenic signaling pathways such as mitogen-activated protein kinase (MAPK) [23], phosphatidylinositol-4,5-bisphosphate 3-kinase (PI3K)-protein kinase B (PKB, AKT) [24], NOTCH receptors (NOTCH1, 2, 3, and 4), NF-κB (nuclear factor kappa B), and Janus kinase (JAK)-signal transducer and activator of transcription (STAT) (JAK-STAT) have been identified in inflammatory conditions [25]. In addition, the characterization of the roles of a variety of angiogenic mediators in re-vascularizing the ischemic myocardium has been the focus of two decades of preclinical research, including vascular endothelial growth factor (VEGF), fibroblast growth factor, hepatocyte growth factor.

Although the reports on this front are very encouraging, the development of efficiently functioning collateral vessels in the ischemic heart still faces a multifaceted challenge, including choosing route of delivery, dosing levels, relevant animal models, duration of treatment, and assessment of phenotype for efficacy [26], which reflect the urgent need to improve our understanding of the expression and regulation of angiogenic factors in cells and tissues in physiological and pathological settings. In this study, we address several fundamental questions including, first, whether tissues have different angiogenic potentials (APs) under physiological conditions; second, what the determining factors for the tissue AP are; and third, how angiogenesis is regulated in different diseases, especially in cancer and myocardial infarction. In this study, we hypothesized that in physiological conditions, tissues have different APs and programs, and in pathological situations, inflammation and immunosuppression regulate ischemic angiogenesis depending on disease/inflammation settings. To examine this hypothesis, we took a panoramic profiling on the expression of 163 angiogenic genes in healthy and diseased human and mouse tissues including 26 transcription factors (TRs), 64 growth factors and receptors (G/Rs), 27 cytokines and chemokines (C/Cs), and 46 proteases and inhibitors (P/Is). We found that, first, the human heart, muscle, eye, lymph node, and pancreas have the highest tissue APs; second, tissue APs may be regulated by cell oxygen sensors PHD2 and HIF1B, VEGF pathway component VEGFB, and stem cell master gene SOX2; third, angiogenesis is differentially regulated in various cancers, chronic metabolic diseases, and autoimmune diseases; and fourth, thrombus-derived leukocytes exhibit more EC-specific angiogenic makers than peripheral blood leukocytes do in acute coronary syndrome patients, which suggests thrombus-derived leukocytes may phenotypically switch into EC-like angiogenic cells to directly promote angiogenesis. Our new findings will improve the current understanding of angiogenesis in healthy and diseased settings and will eventually lead to novel therapeutics for ischemic diseases and cancers.

## Methods

### Tissue expression profiles of angiogenic genes in physiological conditions

**Fig. 1:**
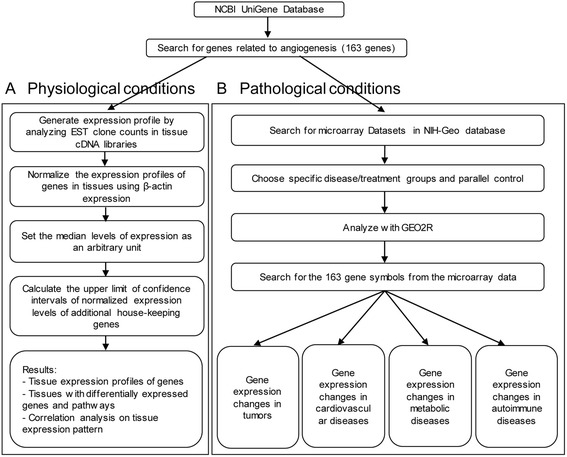
Flow chart of database mining strategy and two parts of data organization. **a** The strategy in generating tissue expression profile of angiogenic genes in physiological conditions. **b** The strategy in analysis of angiogenic gene changes in pathological conditions

**Fig. 2:**
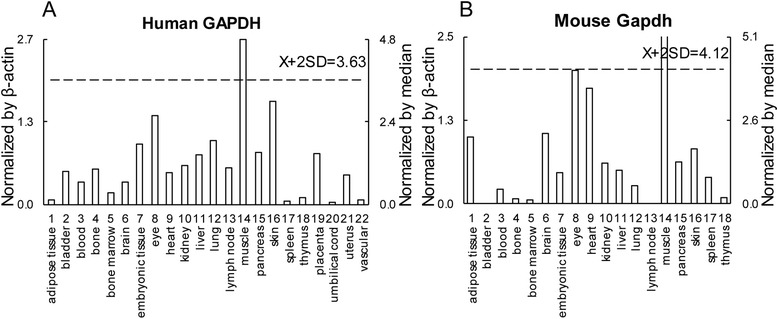
Method of analyzing tissue EST profile. **a** Data presentation format as presented in *X*-axis and *Y*-axis and tissue order is applied to all the genes examined. As an example, the gene expression profile of human house-keeping gene glyceraldehyde-3-phosphate dehydrogenase (GAPDH Hs.544577) in 22 human tissues is shown. The gene expression data was normalized by the β-actin (Hs. 520640) expression data from the same tissue, which is presented on the left *Y*-axis. The arbitrary unit for comparing gene expression level among all tissues was generated by normalizing the gene expression data (after normalization by β-actin) in the tissue with the median expression level among all the tissues, which is presented on the right *Y*-axis. In order to define confidence interval for statistically high expression level of a given gene, we calculated the mean of confidence intervals [mean (*X*) + 2 × standard deviations (SD) = 3.63] of three randomly chosen house-keeping genes including GAPDH, hypoxanthine phosphoribosyltransferase 1 (HPRT1 Hs.412707), and glucuronidase, beta (GUSB Hs.255230). The expression variations of given genes in tissues, when they were larger than 3.63-fold, were defined as the high expression levels with statistical significance (the right *Y*-axis). In this example, GAPDH is highly expressed in muscle. **b** Same strategy applies to mouse genes among 18 tissues. The mean of [X + 2 × SD = 4.12] was determined as the confidence interval for mouse genes expression

As reported in our previous publications [8], we developed an experiment-generated data mining strategy (Fig. 1a) to analyze the messenger RNA (mRNA) expression of 163 of the most relevant angiogenic genes (Additional file 1: Table S1) in various human and mouse tissues based on literature. Expressed sequence tags (ESTs) from the UniGene Library in National Center of Biotechnology Information (NCBI) [27] were used to determine mRNA expression levels in tissues. Transcripts per million (TPM) of genes of interest were normalized with those of house-keeping gene β-actin in each given tissue for comparison among all tissues. These normalized values were then normalized with the median of all tissues to calculate the arbitrary units of gene expression for comparison among all genes (if the median value is 0, we would recalculate the median of tissues with TPM > 0). A confidence interval of the expression variation of house-keeping genes was generated by calculating the mean plus two times that of the standard deviations of the arbitrary units of three randomly selected house-keeping genes (GAPDH, HPRT1, and GUSB) normalized by β-actin in the given tissues (Fig. 2). If the expression variation of a given gene in one tissue was larger than the upper limit of the confidence interval of the house-keeping genes, the expression level of the gene in this tissue was considered high with statistical significance.

### Expression changes of angiogenic genes in pathological conditions

Gene expression profiles were collected from microarray datasets in NIH-GEO database (Fig. 1b). Specific samples were chosen as disease or treatment groups to compare with their parallel controls by GEO2R [28]. The number of samples was always greater than three. By searching for the gene symbols from the microarray data, we selected the genes in our list of 163 angiogenic genes with significant expression changes (*p* < 0.05). The genes of which the expression changes were greater than or equal to 2-fold were defined as the upregulated genes, while genes with their expression changes less than or equal to 0.5-fold were defined as downregulated ones.

### Ingenuity pathway analysis (IPA)

Molecular function types of 163 angiogenic genes were categorized based on IPA database [29] (Additional file 1: Table S1). Functional interactions and potential physical interactions between molecules were illustrated using IPA Path Designer (Fig. 4a and Fig. 6b).

### Protein subcellular location

The subcellular localization of protein was determined using two widely used protein intracellular localization databases [30], namely COMPARTMENTS subcellular location database [31] and UniProtKB/Swiss-Prot location database (European Bioinformatics Institute). Details can be found in our previous publication [10].

## Results

### The heart, muscle, eye, lymph node, and pancreas are among the tissues with the highest APs in humans

**Fig. 3:**
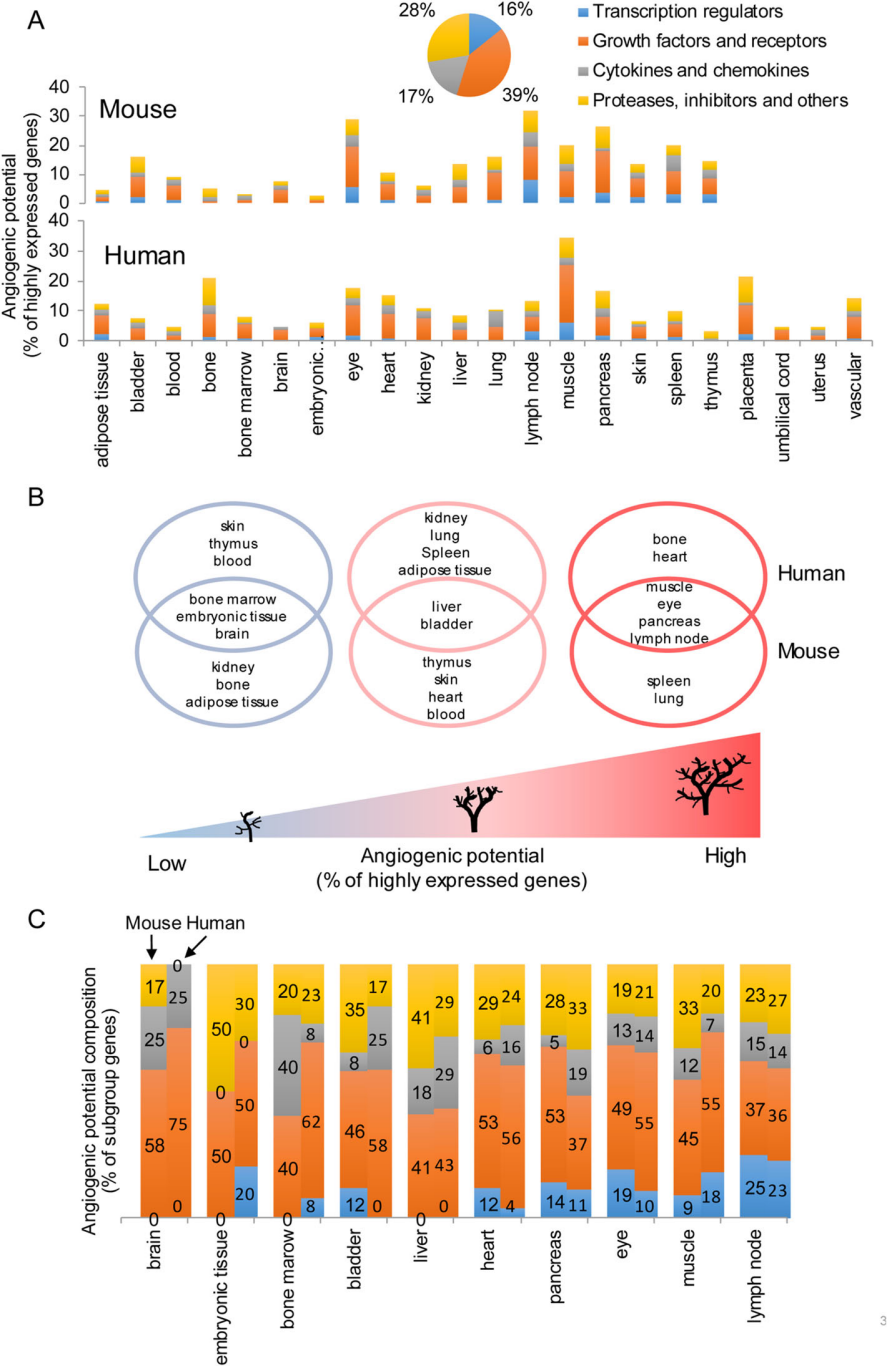
The human heart, muscle, lymph node, eye, and pancreas are among the tissues with the highest angiogenic potentials. **a** Percentage of highly expressed genes among 18 mouse tissues and 22 human tissues indicating angiogenic potentials. We analyzed 163 genes related to angiogenesis, including 26 transcription regulators (16%), 64 growth factors and receptors (39%), 27 cytokines and chemokines (17%), and 46 proteases, inhibitors and others (28%). **b** Three tiers of tissues based on angiogenic potentials among 18 common tissues in human and mouse. The highest six tissues in each species are considered with high angiogenic potential, then the middle six tissues, and the lowest six tissues with low angiogenic potential. **c** Comparison of contributors for angiogenic potentials between human and mouse. The heart and nine overlapped tissues in Fig. 3b are compared for the composition of highly expressed genes in terms of the four groups of angiogenic genes. In each tissue, the *right bar* stands for human, and the *left bar* stands for mouse

We hypothesized that under physiological conditions, tissues undergo different patterns of angiogenesis, which are regulated based on their needs for oxygen and nutrients [32]. To examine this hypothesis, an experiment-generated database mining strategy that we developed (Fig. 2) [8] was used to examine experimentally verified expression profiles of mRNA transcripts of 163 angiogenic genes (Additional file 1: Table S1) in NCBI-UniGene database among 22 human tissues and 18 mouse tissues (fewer mouse tissues were examined due to the fact that gene expression data for the four tissue counterparts in mice were not available in the database). Based on the functional modes of angiogenic genes, they were categorized into four groups based on molecular types in IPA (TRs, GF/Rs, C/Cs, and P/Is). Tissues that had more genes with high expression levels were considered as having higher APs in preparation for physiological changes like wound healing and pathological challenges like ischemia and inflammation. We found that humans and mice have different tissue patterns in terms of AP (Fig. 3a), suggesting that specific tissue AP has been acquired by human tissues as a functional adaptation during evolution. Interestingly, muscle in human has the highest AP, which suggests that sufficient blood supply is highly significant for muscle function, tissue remodeling, and human survival throughout evolution. Among the 18 common tissues shared in humans and mice, muscle, eye, pancreas, and lymph node are the tissues with the highest APs (Fig. 3b). The heart has high AP in humans and moderate AP in mice. We then compared the composition (percentages of four groups) of highly expressed genes among heart and the nine other tissues shared by humans and mice in Fig. 3c. Again, we find the differences of composition patterns in these tissues between humans and mice. Interestingly, APs are positively correlated with the percentages of TRs among highly expressed genes, but not GF/Rs, C/Cs, or P/Is, emphasizing that TRs are the master genes in regulating tissue APs. These results suggest that under physiological conditions, the muscle, eye, lymph node, pancreas, and human heart have already been conditioned with high APs enabling their recovery from pathophysiological insults like wound healing, ischemia, and inflammation.

### Tissues with high angiogenic potentials are substantiated with more active angiogenic pathways and angiogenic cytokine/chemokine responses under physiological conditions

Pathological angiogenesis often involves inflammatory conditions such as tumor angiogenesis and ischemia-induced angiogenesis [25]. As aforementioned, the five most relevant signaling pathways are activated under chronic inflammation, MAPK, PI3K-AKT, NOTCH, NF-κB, and JAK-STAT. We also included two key EC-specific pathways regulating angiogenesis, HIF-VEGF and angiopoietin-tyrosine kinase with immunoglobulin-like and EGF-like domains (ANG-TIE) [33]. Based on the method we developed (Fig. 2), key molecules in each signaling pathway that are most relevant to angiogenesis were examined to determine the activity of each pathway under physiological conditions (Table 1). We determined that if tissues have more than or equal to (≥) 1/3 of pathway-specific angiogenic genes (listed in Table 1) that are highly expressed, these pathways are considered active in those tissues. Similarly, if tissues have more than two active signaling pathways out of the seven pathways, these tissues are considered as having active angiogenic signaling. As shown in Table 2, we found that the human lymph node and muscle are the two tissues having more active global angiogenic signaling in humans than other tissues. A lymph node has two important active pathways, NF-κB and JAK-STAT, which respond to inflammatory stimuli in both humans and mice, while muscle has two key EC-specific active pathways, HIF-VEGF and ANG-TIE, in humans. In mice, the eye, lymph node, spleen, and thymus have more active angiogenic signaling than other tissues. Interestingly, in both humans and mice, immune organs tend to have more active pathways responding to inflammation than others, whereas non-immune organs such as the muscle, eye, heart, and pancreas have more active EC-specific pathways.

**Table 1:** Seven pro-angiogenic pathways are included in this study

Pathways	ID	Number of genes	Gene symbol
MAPK	(1)	6	ETS1 JUN MAPK1 MAPK8 MAPK14 MAP4K4
PI3K-AKT	(2)	8	AKT1 FOXO1 FOXO3 PIK3CA PIK3CB PIK3CD PIK3CG PTEN
NOTCH	(3)	10	DLL1 DLL3 DLL4 JAG1 JAG2 NOTCH1 NOTCH2 NOTCH3 NOTCH4 RBPJ
NF-κB	(4)	5	NFKB1 NFKB2 REL RELA RELB
JAK-STAT	(5)	4	CISH JAK2 STAT1 STAT3
HIF-VEGF	(6)	14	ARNT EGLN1 EPAS1 FIGF FLT1 FLT4 HIF1A KDR NRP1 NRP2 PGF VEGFA VEGFB VEGFC
ANG-TIE	(7)	6	ANGPT1 ANGPT2 ANGPTL3 ANGPTL4 TEK TIE1

**Table 2:**
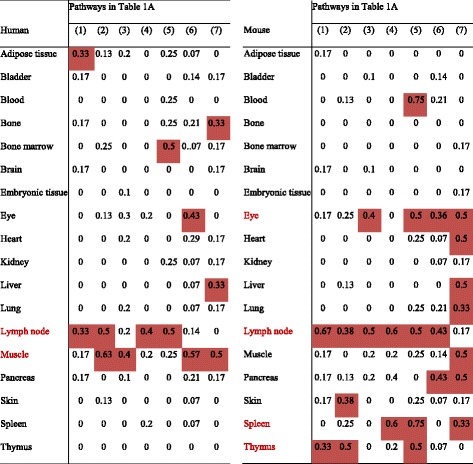
Muscle and lymph node have more active angiogenic pathways in humans: angiogenic pathway expression profiles in 18 common human and mouse tissues

Number in the table stands for the percentage of genes being highly expressed in a specific pathway. Pathways with more than or equal to 1/3 of genes being highly expressed in individual tissue are considered as active pathways (highlighted in red). Tissues with more than two among the seven pathways being active are considered tissues with high angiogenic potentials (text is in red)

Immunotherapy has been developed to treat chronic inflammatory diseases. Thus, we also examined the expression levels of 27 C/Cs, which are most relevant to angiogenesis [34], in different tissues under physiological conditions. Based on the C/Cs roles in modulating angiogenesis, these C/Cs were classified into three categories, pro-angiogenic, anti-angiogenic, and bi-functional (Tables 3 and 4). C/Cs highly expressed in more than median numbers of tissues among all are considered as abundant C/Cs. We found that human CCL5, IL-15, and IL-6 are among the most abundant pro-angiogenic C/Cs in tissues. In contrast, CXCL9 and CXCL14 are the most abundant anti-angiogenic C/Cs. IL-18 is the most abundant human bi-functional C/C. In mice, Ccl2 and Il-15 are the most abundant pro-angiogenic C/Cs; Cxcl11, Cxcl14, and Pf4 are the most abundant anti-angiogenic C/Cs; and Cxcl16 is the most abundant bi-functional C/C. We consider that tissues with more than median numbers of C/Cs being highly expressed among all have high angiogenic C/C responses. We found that the human heart, muscle, eye, pancreas, bone, kidney, liver, and lung are the tissues more likely to develop high angiogenic C/C responses (Table 3); while the mouse eye, lymph node, muscle, spleen, liver, and thymus are the tissues with high angiogenic C/C responses (Table 4). Interestingly, we also found that in humans, more pro-angiogenic C/Cs are highly expressed, while mice have more anti-angiogenic C/Cs highly expressed. This may explain the potential differences between human diseases and mouse models studying angiogenesis.

**Table 3:**
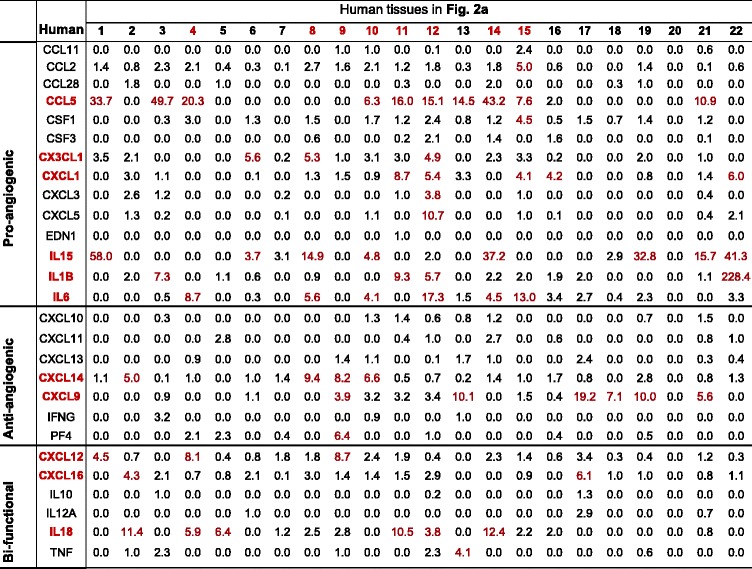
Cytokines/chemokines expression profile in human tissues

IDs for 22 human tissues are the same as Fig. 2a. mRNA relative expression levels of C/Cs higher than threshold are texted red. C/Cs highly expressed in more than the median number of tissues among all are considered as abundant C/Cs (red text). Tissues with more than median number of C/Cs being highly expressed among all are considered as tissues with high angiogenic responses (red text)

**Table 4:**
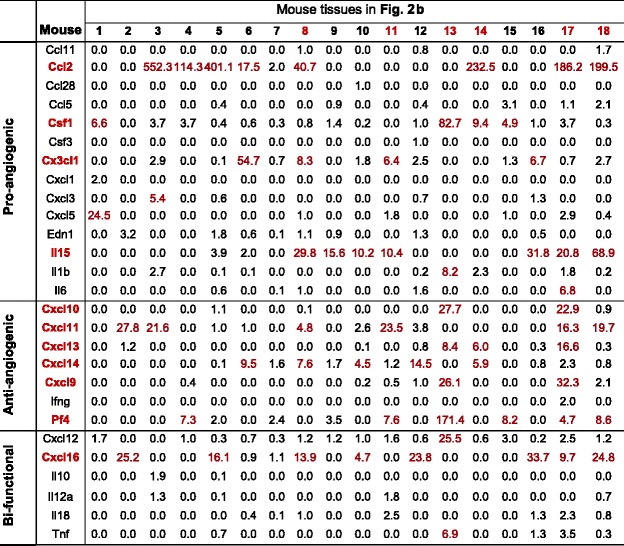
Cytokines/chemokines expression profile in mouse tissues

IDs for 18 mouse tissues are the same as Fig. 2b. mRNA relative expression levels of C/Cs higher than threshold are texted red. C/Cs highly expressed in more than the median number of tissues among all are considered as abundant C/Cs (red text). Tissues with more than median number of C/Cs being highly expressed among all are considered as tissues with high angiogenic responses (red text)

Taken together, these results suggest that the tissues with high APs based on tissue expression of 163 angiogenic genes also have more active pro-angiogenic pathways and stronger C/Cs responses; these angiogenic C/Cs probably are the mediators signaled via the seven angiogenic pathways.

### Inflammatory transcription regulators (TRs) dominate regulation of all angiogenic cytokines/chemokines (C/Cs); homeostatic TRs regulate all to a lower extent, while endothelial cell-specific TRs mainly regulate pro-angiogenic and bi-functional C/Cs

**Fig. 4:**
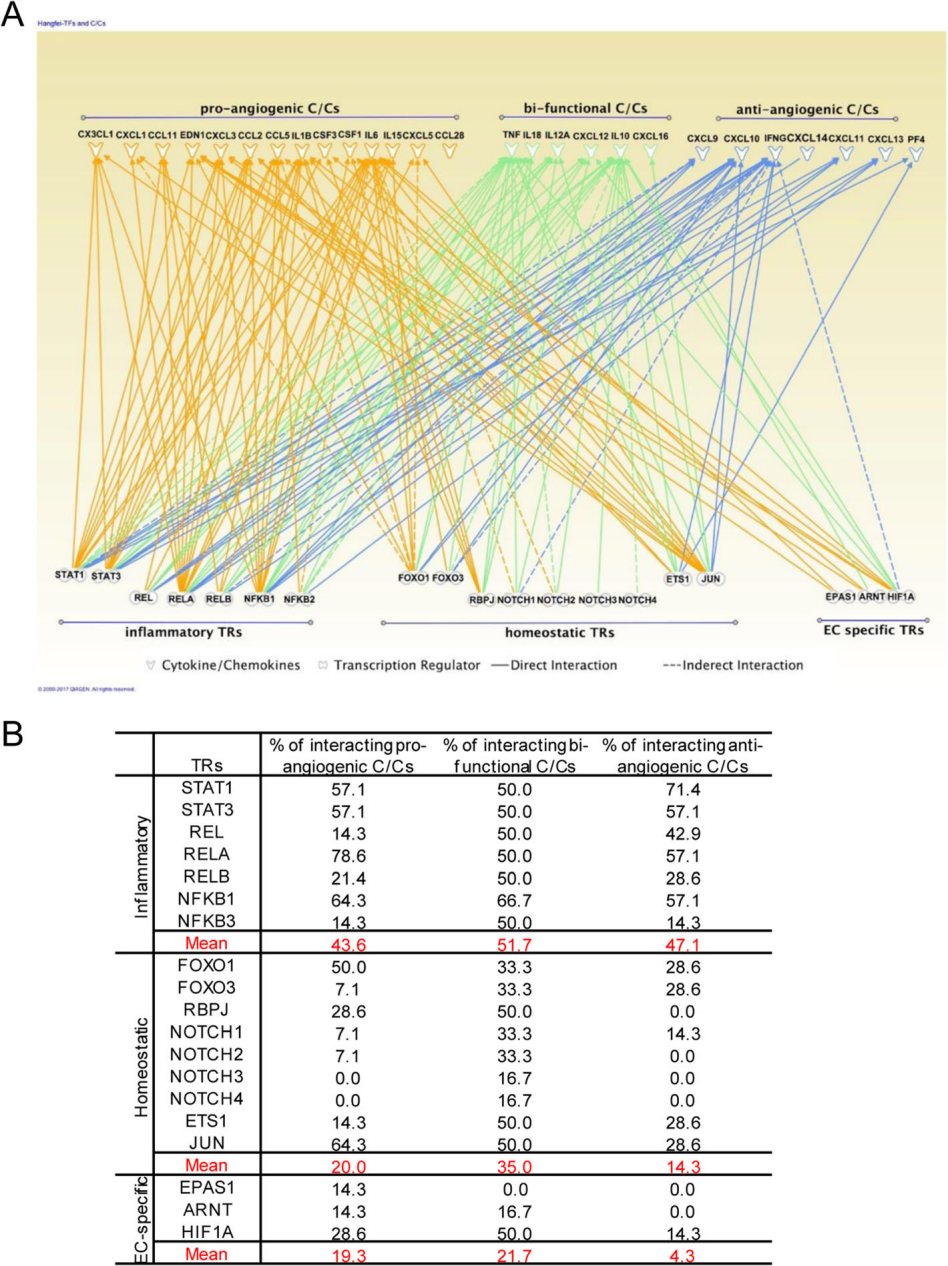
Transcription regulators differentially mediate angiogenic cytokines/chemokines. **a** Functional interactions and potential physical interactions between TRs and C/Cs were illustrated using IPA Path Designer. **b** Summary of TR-regulated C/Cs in three categories

We found that TRs serve as master genes in determining tissue APs in physiological conditions (Fig. 3c). To determine how these seven angiogenic pathways regulate angiogenic C/Cs under pathophysiological conditions, we used the IPA Path Designer to illustrate the interactions between 19 TRs in the seven pro-angiogenic pathways and 27 angiogenic C/Cs based on published literatures (Fig. 4a). We found three major groups of TRs that regulate angiogenesis differentially in various pathophysiological settings: *first*, inflammatory TRs [35] are the main regulators of all three groups of C/Cs, and on average, each inflammatory TR can regulate 43.6% of pro-angiogenic C/Cs, 47.1% of anti-angiogenic C/Cs, and 51.7% of bi-functional C/Cs, suggesting that inflammatory TRs may function differently in different phases of inflammation; *second*, homeostatic TRs can also regulate all three groups of angiogenic C/Cs, albeit to a lesser extent compared to inflammatory TRs (on average, each TR can regulate 20.0% of pro-angiogenic, 14.3% of anti-angiogenic, and 35.0% of bi-functional C/Cs); and *third*, EC-specific TRs in HIF-VEGF pathway mainly regulate pro-angiogenic C/Cs and bi-functional C/Cs (on average, each TR can regulate 19.3% of pro-angiogenic, 4.3% of anti-angiogenic, and 21.7% of bi-functional C/Cs) (Fig. 4b). These results demonstrated that pro-angiogenic TRs are specifically regulating angiogenic C/Cs. Temporally, during acute ischemic conditions, inflammatory regulators initially dominate the local environment. After intense inflammation fades, tissue repair and regeneration, including angiogenesis, can be eventually observed [36]. Thus, inflammatory TRs may regulate different angiogenic C/Cs in angiogenic cells during the mobilizing phase and vessel-building phase during pathogenesis.

### APs among human tissues are positively correlated with tissue expression levels of cell oxygen sensors PHD2 and HIF1B, VEGF pathway component VEGFB, and stem cell master gene SOX2

**Fig. 5:**
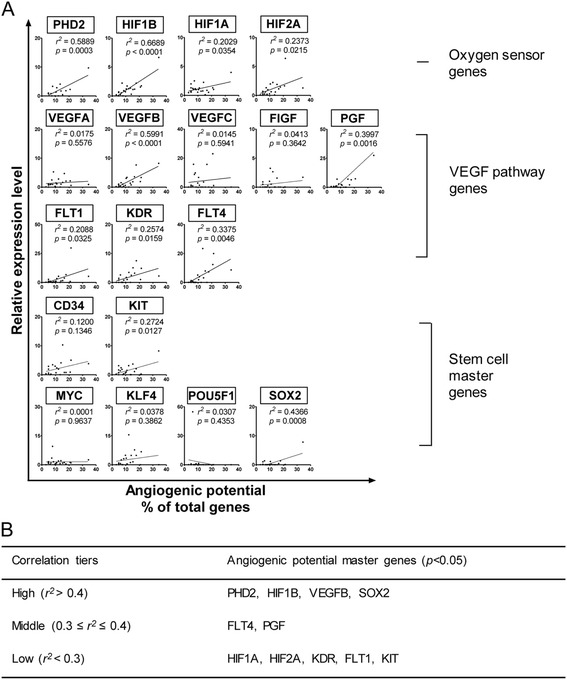
Genes that determine angiogenic potentials in human tissues. **a** Correlation of mRNA relative expression levels of specific genes with angiogenic potential. Simple linear regression was applied to the mRNA relative expression levels (*Y*-axis) against angiogenic potentials (*X*-axis) in total 163 genes. **b** Three tiers of master genes determining angiogenic potential. Eleven genes with *p <* 0.05 were categorized into three tiers based on *r* ^*2*^ value

To address the question of what factors could determine tissue APs, we hypothesized that tissue APs are positively correlated with the tissue expression levels of cell oxygen sensors. To examine this hypothesis, simple linear regression analyses were performed by plotting mRNA relative expression levels of PHD2 (EGLN1), HIF1A, HIF2A (EPAS1), and HIF1B (ARNT) against the percentages of highly expressed genes in each tissue (based on total 163 genes). We found that the mRNA relative expression levels of all four genes could indicate tissue APs in humans (Fig. 5a). Interestingly, expression levels of PHD1 (EGLN2) and PHD3 (EGLN3) did not correlate with tissue APs (data not shown). Since VEGF pathway components are key effectors in mediating angiogenesis [3], in addition to the oxygen sensors, we also examined the correlation of VEGFs/VEGFRs mRNA expression levels with tissue APs (Fig. 5a). We found that the expression levels of VEGFB, PGF, VEGFR1 (FLT1), VEGFR2 (KDR), and VEGFR3 (FLT4) are positively correlated with tissue APs. Since tissue repair and regeneration processes largely rely on angiogenesis, we also determined whether the expression of stem cell master genes including CD34, KIT, MYC, KLF4, OCT4 (POUSF1), and SOX2 are positively correlated with tissue APs. Of note, MYC, KLF4, OCT4, and SOX2 are the induced pluripotent stem (IPS) cell transcription factors or Yamanaka’s re-programming transcription factors [37]. Interestingly, the results showed that among the seven stem cell master genes examined, the expression levels of KIT (*r* ^2^ = 0.2724; *p* < 0.05) and SOX2 (*r* ^2^ > 0.4; *p* < 0.05) are positively correlated with tissue APs (Fig. 5b). Based on the different correlation factor *r* ^*2*^between the expression levels of angiogenic genes and tissue APs, we categorized three types of angiogenic regulators into three levels of correlation factor *r* ^*2*^, high, middle, and low (Fig. 5b). The results suggest that the APs among human tissues have high correlation with the tissue expression levels of cell oxygen sensors PHD2 and HIF1B, VEGF pathway component VEGFB, and stem cell master gene SOX2, moderate correlation with the tissue expression levels of PGF and FLT4, and low correlation with the tissue expression levels of HIF1A, HIF2A, KDR, FLT1, and KIT.

To further understand how these angiogenic regulators indicate tissue APs, we categorized 163 genes into four groups of angiogenic genes (TRs, GF/Rs, C/Cs, and P/Is) and found that (1) the expression of VEGFB positively correlates with APs of all four groups of genes; (2) the expression levels of HIF1B and PHD2 positively correlate with APs of TRs, GF/Rs, and P/Is; and (3) the expression level of SOX2 positively correlates with APs of TRs and GF/Rs (Additional file 1: Figure S1). These results suggest that different AP master genes are associated with the expression of specific groups of angiogenic genes in regulating tissue angiogenesis.

### Cancers in digestive system tend to have increased angiogenesis dominated by EC-specific pro-angiogenic pathways, while lung cancer and prostate cancer have significantly decreased angiogenesis

Anti-angiogenic therapy has been suggested as an approach to treat cancers decades ago [32]. The underlying rationale, stating that solid tumor expansion is dependent on blood supply, has been universally accepted. Many drugs targeting angiogenesis have been developed or are under clinical trials. However, the clinical outcomes from patients treated with anti-angiogenic drugs are less satisfactory than expected [38]. There are no good mechanistic explanations on why this discrepancy exists. We hypothesize that cancers from different tissues have distinct angiogenic pathways and C/C responses. To test this issue, we examined seven most relevant pro-angiogenic pathways including MAPK, PI3K-AKT, NOTCH, NF-κB, JAK-STAT, HIF-VEGF, and ANG-TIE using the method that we developed (Fig. 1b). We searched angiogenic gene expression data in 11 GSE datasets collected in the NIH-GEO Database (microarray experimental data) on most common cancers (six types of cancers in digestive system, three types of cancers in reproductive system, lung cancer in respiratory system, and lymphoma in lymphoid system) to better understand the molecular mechanisms underlying cancer/tumor angiogenesis (Table 5). In each GSE dataset, we compared the mRNA expression levels in cancer/tumor tissues to their adjacent normal tissues. We found that digestive cancers have significant gene expression changes enriched in HIF-VEGF and ANG-TIE pathways, especially in colorectal, gastric, and intrahepatic cancers, while in reproductive cancers and lung cancer, most of the pathways are regulated. Interestingly, pancreatic cancer did not show any changes in these seven pathways, suggesting that the high AP in pancreas (Fig. 3b) are equipped to match the high demand for angiogenesis in both physiological and tumor/cancer pathological conditions. As we know, most cancers have increased angiogenesis to supply blood and nutrition for cancer/tumor growth [38]. Surprisingly, here we found that prostate cancer, lung cancer, and lymphoma show significantly decreased gene expression among most changed pathways. This could be explained by sufficient oxygen supply for cancer cells in the lung, T cell-mediated immune tolerant/anti-inflammatory environment in the lung [39], and in circulating prostate cancer cells [40]. Many C/Cs play indispensable roles in communicating between immune cells, vascular cells, as well as tumor cells in regulating angiogenesis [25]. Thus, we further looked into angiogenesis-regulatory C/Cs in different disease conditions. We found that most digestive cancers and ovarian cancer have both pro- and anti-angiogenic C/Cs being upregulated, indicating that the C/Cs system is in a “fighting” mode (Table 6). However, in prostate cancer and lung cancer, where we have shown that they present an anti-angiogenic environment in the pathway analysis (Table 5), pro-angiogenic C/Cs are significantly downregulated and anti-angiogenic C/Cs are upregulated (Table 6). These results suggested that angiogenic pathways in various cancers are differentially regulated in different tissue environments and pathological conditions. In order to specifically target key molecules during the disease conditions, we would need more precise and accessible detection systems in identifying the personalized angiogenic gene expression profiles for timely and accurate anti-angiogenic treatments for cancer patients.

**Table 5:**
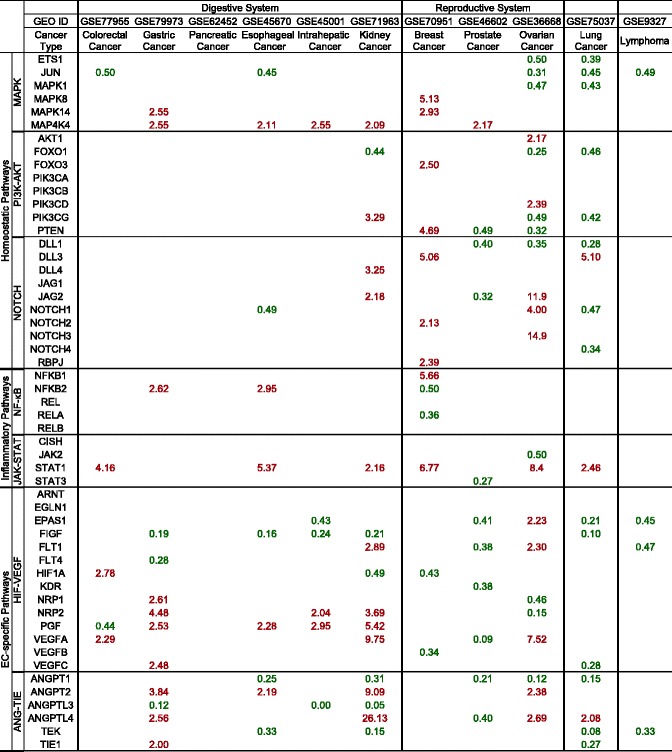
Gene expression changes of pro-angiogenic pathways in cancers

Seven master pro-angiogenic pathways including MAPK, PI3K-AKT, NOTCH, NF-κB, JAK-STAT, HIF-VEGF, and ANG-TIE are investigated using the method previously developed (Fig. 1a). We compared gene expression levels in tumor tissues to adjacent normal tissues among six types of cancers in the digestive system, three types of cancers in the reproductive system, lung cancer, and lymphoma as shown. Genes with significant expression changes (*p <* 0.05) are shown here. Red text stands for upregulation (FC ≥ 2), and green text stands for downregulation (FC ≤ 0.5)

**Table 6:**
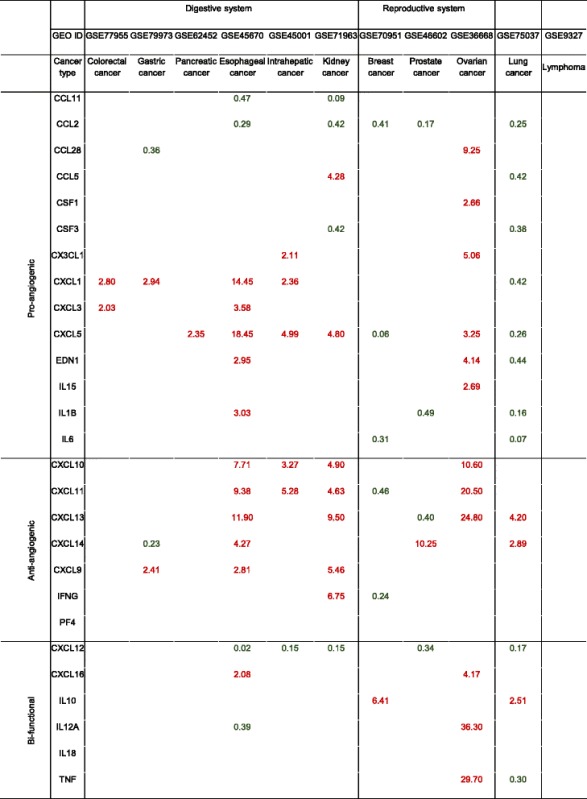
Gene expression changes of cytokines/chemokines in cancers

Twenty-seven cytokines/chemokines are investigated using the method we developed (Fig. 1b). We compared gene expression level in tumor tissues to adjacent normal tissues among six types of cancers in the digestive system, three types of cancers in the reproductive system, lung cancer, and lymphoma as shown. Genes with significant expression changes (*p <* 0.05) are shown here. Red text stands for upregulation (FC ≥ 2), and green text stands for downregulation (FC ≤ 0.5)

### Pro-angiogenic pathways are significantly upregulated in thrombus-derived leukocytes in patients with acute coronary syndrome, retinopathy, and rheumatoid arthritis; various subcellular-localized upregulated angiogenic proteins in thrombus leukocytes are endothelial cell-specific

**Fig. 6:**
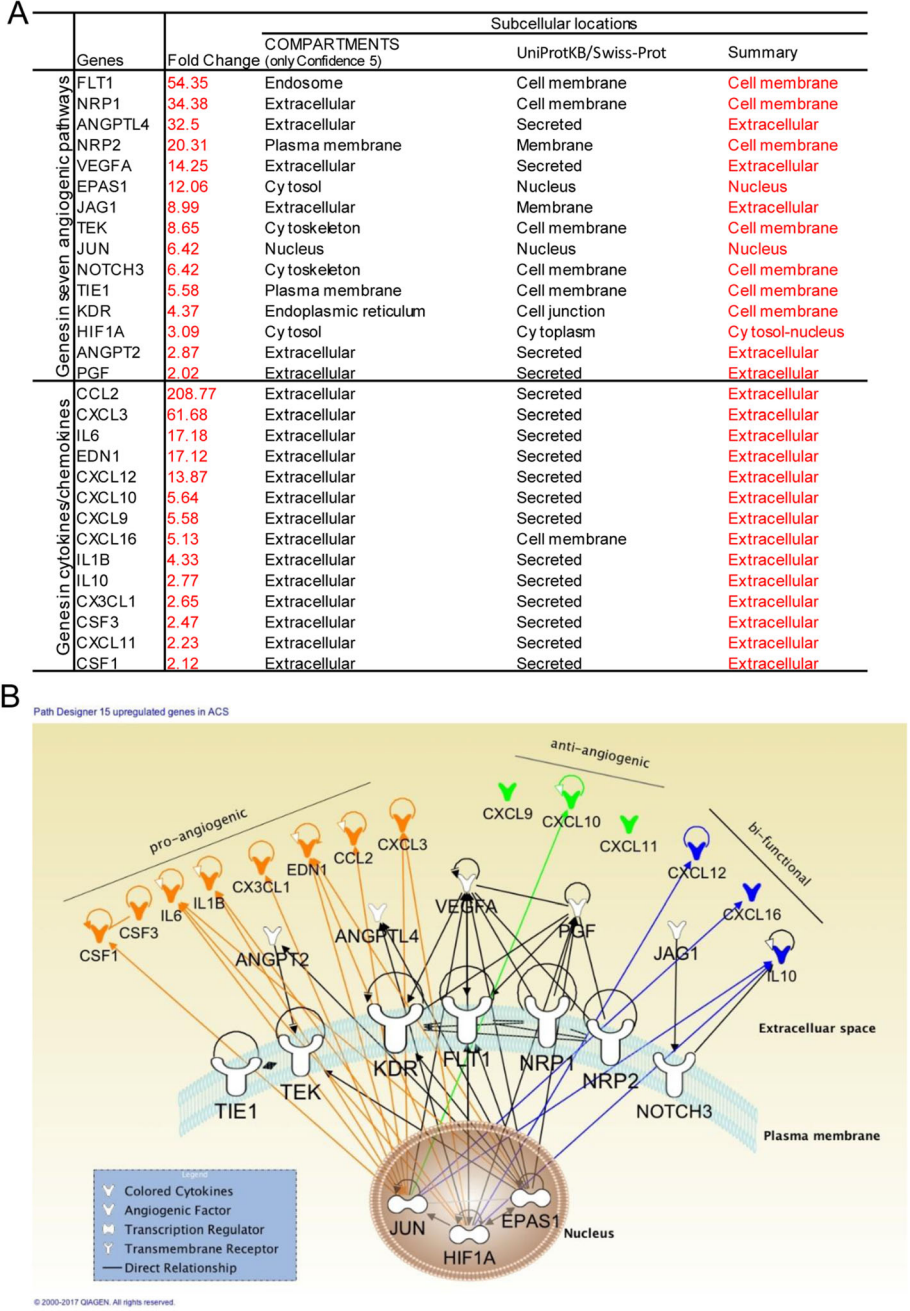
Thrombus leukocytes phenotypically switch into endothelial cell-like angiogenic cell in acute coronary syndrome patients. **a** Subcellular locations of upregulated genes among different pathways and cytokines/chemokines in acute coronary artery disease dataset GSE19339 (Tables 6 and 7). **b** Molecular interactions between upregulated genes, generated by using IPA Path Designer

Angiogenesis in thrombus has been shown to play a key role in facilitating thrombus resolution [41, 42]. Hypoxia and HIF1A are induced in the naturally resolving thrombus and correlate with increased angiogenic factor expression. Upregulation of HIF1A enhances thrombus resolution and angiogenesis [43]. Leukocyte infiltration is one of the hallmarks during this process. To examine an important question of whether immune cells can directly promote angiogenesis, we compared the angiogenic gene expression profile of leukocytes isolated from thrombi from the plaque of acute coronary syndrome (ACS) patients with that in circulating blood leukocytes. We found that in ACS (unpublished GEO microarray dataset GSE19339, deposited on Dec. 4, 2009, and updated on Feb. 24, 2017), thrombus-derived white blood cells showed significant increase of angiogenic pathways with as many as 15 angiogenic regulators compared to peripheral leukocytes (Table 7). Although the research paper associated with this GEO dataset has not been published from Dr. Luscher’s team in the University Hospital Zurich, Switzerland, based on similar publications on isolation of Tregs [44], CD14^+^ monocytes and CD66B^+^ granulocytes [45] in coronary thrombi of patients with ACS and microarray profiling [46] from this highly productive team, we were fully convinced that the high quality of GSE19339 dataset and related technologies in coronary thrombus leukocyte isolation and microarray profiling are trustable. In addition, to determine the functional significance of upregulated genes, we examined the subcellular localization of the 15 upregulated genes including FLT1, NRP1, ANGPTL4, NRP2, VEGFA, EPAS1, JAG1, TEK, JUN, NOTCH3, TIE1, KDR, HIF1A, ANGPT2, and PGF using two highly reliable databases (COMPARTMENTS subcellular location database and UniProtKB/Swiss-Prot location database) as we reported [10] (Fig. 6a). As shown in Table 7, we found that among the upregulated genes, seven genes encode EC-surface receptors including FLT1 (vascular endothelial growth factor receptor 1), NRP1 (vascular endothelial cell growth factor 165 receptor), NRP2 (vascular endothelial cell growth factor 165 receptor 2), KDR (cell surface receptor for VEGFA, VEGFC and VEGFD) [47], TIE1, TEK, and NOTCH3. Moreover, EPAS1 and HIF1A are two transcription factors (Fig. 6a) directly involved in hypoxia-induced gene expression pathway; homeostatic transcription regulator JUN (Fig. 6a) is a subunit of the transcription factor AP-1 that was reported to collaborate with HIF1 to increase gene expression in response to hypoxia [48, 49]. The remaining five molecules upregulated in thrombus-derived leukocytes were all pro-angiogenic factors, including ANGPT2, ANGPTL4, VEGFA, PGF, and JAG1, which we propose can stimulate thrombus leukocytes to phenotypically switch into EC-like angiogenic cells, and thrombus-trapped circulating ECs via an autocrine and paracrine manner, to create a pro-angiogenic niche. However, peripheral blood leukocytes from ACS patients did not show the similar changes compared to healthy control (GSE 61144) (Table 7). We also found that a significant amount of C/Cs in thrombus-derived leukocytes are upregulated (Table 8), including 8 out of 14 pro-angiogenic C/Cs, 3 out of 7 anti-angiogenic ones, and 3 out of 6 bi-functional ones. The information shown in Fig. 6b suggests that the most upregulated C/Cs can be directly regulated by the three upregulated TRs (EPAS1, HIF1A, and JUN). Taken together, these results have demonstrated that thrombus-derived leukocytes exhibit potent pro-angiogenic phenotype by upregulating (i) EC-specific cell surface markers and (ii) hypoxia-induced transcription factor signaling pathways and secreting (iii) angiogenic factors and (iv) angiogenic C/Cs. These findings suggest that thrombus leukocytes promote angiogenesis not only through increasing secretion of angiogenic C/Cs and angiogenic factors but also via phenotypical switching into EC-like angiogenic cells.

**Table 7:**
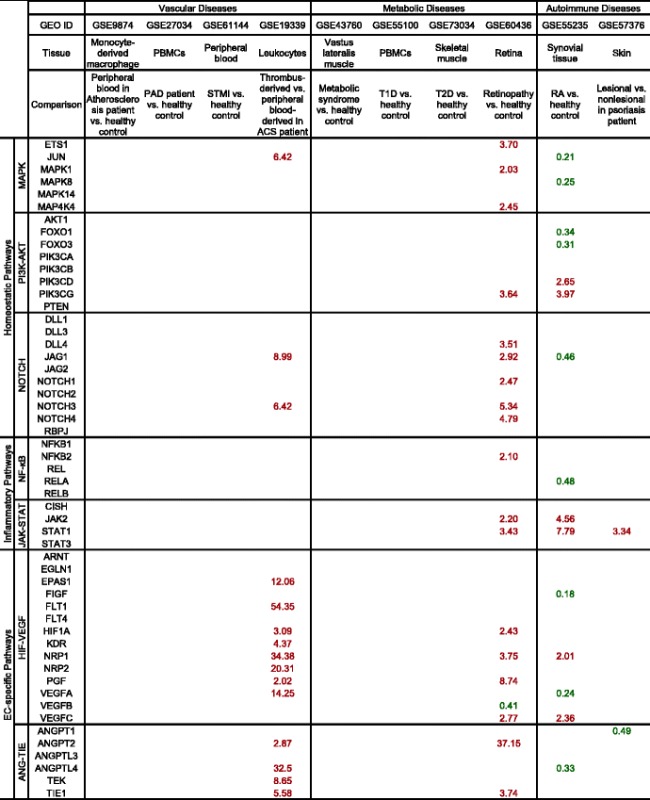
Gene expression changes of pro-angiogenic pathways in vascular diseases, metabolic diseases, and autoimmune diseases

Seven master pro-angiogenic pathways including MAPK, PI3K-AKT, NOTCH, NF-κB, JAK-STAT, HIF-VEGF, and ANG-TIE are investigated using the method we developed (Fig. 1b). We compared gene expression level in different tissues to their parallel controls. Genes with significant expression changes (*p <* 0.05) are shown here. Red text stands for upregulation (FC ≥ 2), and green text stands for downregulation (FC ≤ 0.5)

**Table 8:**
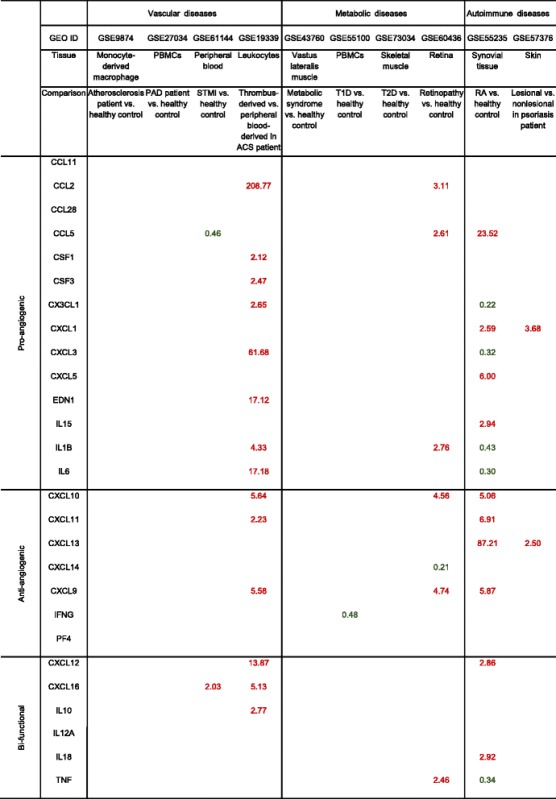
Gene expression changes of cytokines/chemokines in vascular diseases, metabolic diseases, and autoimmune diseases

Twenty-seven cytokines/chemokines are investigated using the method we developed (Fig. 1b). We compared gene expression level in different tissues to their parallel controls. Genes with significant expression changes (*p <* 0.05) are shown here. Red text stands for upregulation (FC ≥ 2), and green text stands for downregulation (FC ≤ 0.5)

We also found that retinopathy and rheumatoid arthritis (RA) have significant changes in many pro-angiogenic pathways. However, in the conditions of chronic inflammation, atherosclerosis, PAD, metabolic syndrome (MS), type 1 diabetes (T1D), and type 2 diabetes (T2D), there are no significant changes in any of these seven pathways (Table 7), suggesting that low-dose chronic inflammation does not always have a strong systemic angiogenic stimulation. We also examined the C/C expression changes among these diseases. We found that a significant number of C/Cs is upregulated in retinopathy and RA. However, in the chronic inflammatory diseases, there are little to no changes in C/C expression (Table 8). Taken together, these results suggest that diseases with active angiogenic pathways also have highly active C/C responses for cell-cell communication.

## Discussion

Significant progress has been made in characterizing factors regulating angiogenic pathways related to physiological and pathological processes [47], which has led to the development of preclinical assessment of anti-angiogenic therapies for cancers and other diseases [50]. However, angiogenic therapies have been less than satisfactory [38]. Thus, the development of efficient therapeutics for various clinical settings for inhibiting cancer/tumor growth and improving angiogenesis for re-gaining blood supply in ischemic heart and ischemic brain areas after stroke still faces multifaceted challenges [26], which reflect the urgent need for improving our understanding of the expression and regulation of angiogenic factors in cells and tissues in physiological and pathological settings. In this study, we examined the expression of 163 angiogenesis-regulatory genes in various tissues in humans and mice in physiological settings and in various pathological processes. We have made the following significant findings: (1) the heart, muscle, eye, lymph node, and pancreas are among the tissues with the highest APs in humans; (2) tissues with high angiogenic potentials have more active angiogenic pathways and angiogenic C/C responses under physiological conditions; (3) inflammatory TRs dominate regulation of all angiogenic C/Cs, and homeostatic TRs regulate all to a lower extent, while endothelial cell-specific TRs mainly regulate pro-angiogenic and bi-functional C/Cs; (4) APs among human tissues are positively correlated (*r* ^*2*^ > 0.4) with tissue expression levels of cell oxygen sensors PHD2 and HIF1B, VEGF pathway component VEGFB, and stem cell master gene SOX2; (5) cancers in digestive system tend to have increased angiogenesis dominated by EC-specific pro-angiogenic pathways, while lung cancer and prostate cancer have significantly decreased angiogenesis; and (6) pro-angiogenic pathways are significantly upregulated in thrombus-derived leukocytes in patients with acute coronary syndrome, retinopathy, and rheumatoid arthritis, and various subcellular-localized upregulated angiogenic proteins in thrombus leukocytes are EC-specific. Our novel findings have provided significant insights into molecular mechanisms in regulating angiogenesis in various tissue physiological and pathological processes.

In determining the master regulators for tissue angiogenesis, two important issues, which are also related to tissue regenerative potential, have never been addressed: *first*, whether various tissues have differences in angiogenesis and *second*, what determines tissue AP if there are differences. As we know, all eukaryotic organisms rely on oxygen to support oxidative phosphorylation for efficient adenosine triphosphate (ATP) production. Therefore, a constant O_2_ supply, maintained by the vascular system in mammals, is critical for proper tissue development, homeostasis, and function. However, the normal physiological O_2_ concentration varies greatly among tissues. For example, arterial blood has a normal partial oxygen pressure (pO_2_) of 13%, the myocardium has a pO_2_ of 10%, and most tissues, including the brain, lung, liver, muscle, and bone marrow, have a pO_2_ of ~5% [51]. Thus, tissue pO_2_ levels cannot directly serve as master regulator of the expression of angiogenic genes and therefore tissue AP. To address these questions, we examined the gene expression levels of 163 angiogenic genes in 22 human tissues and 18 mouse tissues. We have found that in physiological conditions, significant positive correlations exist between APs in various tissues and the tissue expression levels of oxygen sensors PHD2 and HIF1B, VEGF pathway component VEGFB, and stem-ness gene SOX2 (Fig. 5b). Our results on tissue APs support current understanding that PHD2, rather than PHD1 or PHD3, is the critical oxygen sensor in normoxia [52]. HIF1B is constitutively expressed, which binds to HIF1A or HIF2A to activate transcription of hypoxia-responsive genes [53]. When tissues with high APs encounter ischemic conditions, significant downregulation of PHD2 leads to de-suppression of HIF1A and HIF2A, which can bind to highly stably expressed HIF1B, and facilitate these tissues’ initiation of quick and strong angiogenic response. It has been reported that VEGFB binds specifically to VEGFR1, which is complex and context-dependent; and that increased VEGFB expression correlates with cancer stage, tumor multiplicity, and vascular invasion in multiple cancers [54]. Thus, our new finding suggests that VEGFB fits well for the angiogenesis indicator. Of note, SOX2 is one of the IPS cell transcription factors or Yamanaka’s re-programming transcription factors [37] and can be induced in angiogenic pericytes in response to hypoxia [55]. In addition, SOX2 functions with VEGF and drives cancer-initiating stem cells [56, 57]. Our results are the first report showing that tissue expression level of SOX2, but not of other Yamanaka’s re-programming transcription factors including MYC, KLF4, and OCT4, is significantly correlated with tissue APs.

One of the most promising and best studied of anti-angiogenic therapies utilized to date is bevacizumab, a humanized monoclonal antibody against VEGFA. Bevacizumab increases survival when added to chemotherapy in first-line treatment of patients with previously untreated metastatic colorectal cancer. However, the addition of bevacizumab has failed to generate survival advantages for metastatic disease when added to first-line chemotherapy at other sites, including metastatic breast, pancreatic, or ovarian cancer [58, 59]. These results are consistent with our findings that (1) pro-angiogenic pathways in colorectal cancer are more EC-specific, while pro-angiogenic pathways in breast or ovarian cancers are not and (2) pancreatic cancer patients show little to no changes in pro-angiogenic pathways. However, our findings are based on limited datasets in different cancer types. To confirm that EC-specific pathway dominating pro-angiogenic cancers will have better response to anti-VEGF therapy, more robust metadata analyses and experimental studies are needed.

Arterial thrombosis leads to acute ischemic diseases, including myocardial infarction, stroke, and PAD. Thrombus angiogenesis has been shown to play significant roles in promoting thrombus resolution, which may be a potential therapeutic strategy [41]. In addition, anti-angiogenic cancer therapies have been shown to be associated with a significant increase in the risk of arterial thromboembolic events [60] and venous thromboembolic events [42]. A meta-analysis of randomized controlled trials showed that treatment with anti-angiogenic drug bevacizumab might significantly increase the risk of cardiac ischemic events in cancer patients; however, the risk of ischemic stroke with bevacizumab was not significantly different from controls [61]. Our recent reports have shown that inhibition of innate immune sensor caspase-1 improves VEGFR signaling, angiogenesis [62], and neovascularization of progenitor cells after myocardial infarction [16]. Inflammatory and innate immune cells migrated to atherogenic arteries promote the formation of plaques and thrombi or limit tissue damage and facilitate resolution [63] while atherothrombosis, subsequent to plaque rupture or erosion, has prominent features of inflammation in patients with acute coronary syndromes [45]. It was reported that monocytes/macrophages are the most abundant inflammatory cell types of innate immunity in coronary atherothrombosis co-expressing Toll-like receptor 4 [45]. Immune cells, including Tregs, macrophages, monocytes and dendritic cells, have been shown to regulate angiogenesis in different disease settings by providing pro-angiogenic C/Cs and pro-angiogenic niches [64]. However, other roles of thrombus leukocytes, outside of the several reported roles such as triggering thrombosis, causing unstable angina and myocardial infarction, remain unknown. Thus, we attempted to address an important question of whether immune cells can phenotypically switch into EC-like angiogenic cells. We compared the angiogenic gene expression in leukocytes (mainly CD14^+^ monocytes and CD66B^+^ granulocytes) [45] isolated from thrombi from the plaque of ACS patients to that in circulating blood leukocytes. We found the exciting results that as many as 15 important angiogenic regulators and 14 angiogenic C/Cs are significantly upregulated in thrombus leukocytes in comparison to those in peripheral blood leukocytes. The 15 upregulated genes include seven EC-surface receptors for angiogenesis, three hypoxia-response transcription factors, and other important angiogenic secreting regulators. Our results have demonstrated for the first time that thrombus-derived leukocytes can promote angiogenesis not only through secreting angiogenic C/Cs and angiogenic factors but also via phenotypical switching into EC-like angiogenic cells by upregulating EC-specific cell surface markers and hypoxia-induced transcription factor signaling pathways. Our findings suggest thrombus-derived leukocytes may trans-differentiate [65] into EC-like angiogenic cells, which is similar to Dr. Owens’ team’s report of trans-differentiated vascular smooth muscle cell (VSMC)-derived macrophage-like cells in atherosclerotic plaques [66].

Our findings are based on a comprehensive database mining strategy, which allows us to understand angiogenesis in a broad perspective. However, the limitations of this study include (1) only transcriptional expression level was examined in both physiological and pathological conditions and (2) no experimental studies are available to compare with our proposed findings. To confirm these findings, further experiments with genetically modified mouse models and genetic lineage tracing method should be performed.

## Conclusions

**Fig. 7:**
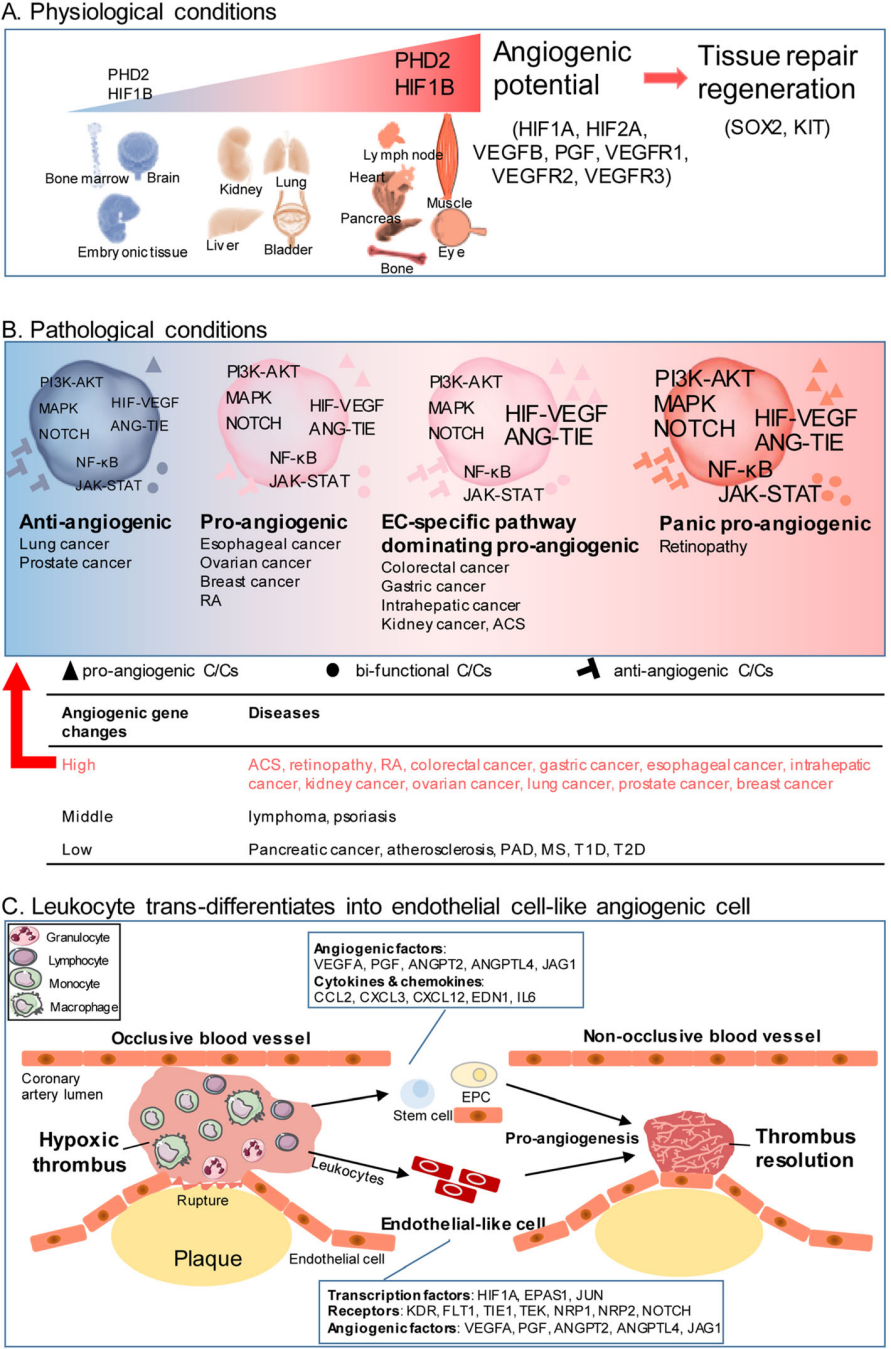
New understanding of tissue-specific and disease-specific angiogenesis. **a** Angiogenic potential in physiological conditions. Master genes for angiogenic potential are as listed. Font size of gene correlates with its mRNA expression level (PHD2 and HIF1B). **b** Angiogenesis in pathological conditions. Font size of pathway name correlates with its gene expression level. Number of C/Cs correlates with their expression level. **c** Thrombus leukocytes may trans-differentiate into endothelial cell-like angiogenic cells in ACS patients

To summarize our findings, we propose the following new working model (Fig. 7): *first*, angiogenic potentials are varied among tissues, which are found in the highest in the heart, muscle, eye, lymph node, and pancreas and in the lowest in bone marrow, skin, thymus, and brain (Fig. 7a); *second*, tissue angiogenic potentials may be regulated by cell oxygen sensors (PHD2, HIF1B, HIF1A, and HIF2A), VEGF pathway components (VEGFB, PGF, and VEGFR1-3), and stem cell master genes (SOX2 and KIT) (Fig. 7a); *third*, angiogenesis is differentially regulated in various cancers, chronic metabolic diseases, and autoimmune diseases (Fig. 7b); and *fourth*, thrombus-derived leukocytes exhibit significantly upregulated EC-specific cell surface markers, hypoxia-induced transcription factor signaling pathways, and secrete angiogenesis factors and C/Cs (Fig. 7c). This also suggests that thrombus leukocytes promote angiogenesis not only through increasing secretion of angiogenic C/Cs and angiogenic factors but also via phenotypical switching into EC-like angiogenic cells. Our novel findings could lead to new development of future therapeutics for regulating tissue-specific angiogenesis, tissue regeneration, and various diseases including cancers and myocardial infarction.

## Additional files

Additional file 1: Figure S1. Correlation of mRNA relative expression levels of specific genes with angiogenic potential in human tissues. Simple linear regression was applied to the mRNA relative expression levels (*Y*-axis) against angiogenic potentials (*X*-axis) in each group of angiogenic genes (transcription regulators, growth factors and receptors, cytokines and chemokines, and proteases, inhibitors, and others). **Table S1.** Summary of 163 genes related to angiogenesis.

## Abbreviations

ACS: Acute coronary syndrome
AKT (PKB): Protein kinase B
ANG: Angiopoietin
AP: Angiogenic potential
ATP: Adenosine triphosphate
C/C: Cytokine and chemokine
EC: Endothelial cell
EPC: Endothelial progenitor cell
EST: Expressed sequence tag
GF/R: Growth factor and receptor
HIF: Hypoxia-induced factor
IL-1β: Interleukin-1β
IL-35: Interleukin-35
IPA: Ingenuity pathway analysis
IPS: Induced pluripotent stem
JAK: Janus kinase
MAPK: Mitogen-activated protein kinase
NCBI: National Center of Biotechnology Information
NF-κB: Nuclear factor kappa B
P/I: Protease, inhibitor and other
PAD: Peripheral artery disease
PBMC: Peripheral blood mononuclear cell
PHD: Hypoxia-inducible factor prolyl hydroxylase
PI3K: Phosphatidylinositol-4,5-bisphosphate 3-kinase
pO_2_: Partial oxygen pressure
RA: Rheumatoid arthritis
STAT: Signal transducer and activator of transcription
STMI: ST segment elevation myocardial infarction
T1D: Type 1 diabetes
T2D: Type 2 diabetes
TIE: Tyrosine kinase with immunoglobulin-like and EGF-like domains
TPM: Transcripts per million
TR: Transcription regulator
Treg: Regulatory T cell
VEGF: Vascular endothelial growth factor
VSMC: Vascular smooth muscle cell
