# Supplementary material for: Thrombus leukocytes exhibit more endothelial cell-specific angiogenic markers than peripheral blood leukocytes do in acute coronary syndrome patients, suggesting a possibility of trans-differentiation: a comprehensive database mining study
Source: J Hematol Oncol. 2017 Mar 23;10:74. doi: 10.1186/s13045-017-0440-0 (PMC5364721; doi:10.1186/s13045-017-0440-0)
Supplement: Supplementary file 1 — Correlation of mRNA relative expression levels of specific genes with angiogenic potential in human tissues. Simple linear regression was applied to the mRNA relative expression levels (Y-axis) against angiogenic potentials (X-axis) in each group of angiogenic genes (transcription regulators, growth factors and receptors, cytokines and chemokines, and proteases, inhibitors, and others). Table S1. Summary of 163 genes related to angiogenesis. [file 13045_2017_440_MOESM1_ESM.docx]

Additional file 1

**Figure S1. Correlation of mRNA relative expression levels of specific genes with angiogenic potentials in humans.** Simple linear regression was applied to the mRNA relative expression levels against angiogenic potentials in four groups of angiogenic genes (transcription regulators, growth factors and receptors, cytokines and chemokines, and proteases, inhibitors and others).

**Table S1. Summary of 163 genes related to angiogenesis**.

| Symbol | Transcription regulators | Human UniGene ID | Mouse UniGene ID | Reference/PubMed ID |
| --- | --- | --- | --- | --- |
| ARNT | transcription regulator | Hs.632446 | Mm.250265 | 9223322 |
| EPAS1 | transcription regulator | Hs.468410 | Mm.1415 | 17589895 |
| ETS1 | transcription regulator | Hs.369438 | Mm.292415 | [25] |
| FOXO1 | transcription regulator | Hs.370666 | Mm.29891 | [25] |
| FOXO3 | transcription regulator | Hs.220950 | Mm.338613 | [25] |
| HAND2 | transcription regulator | Hs.388245 | Mm.430844 | 16849646 |
| HIF1A | transcription regulator | Hs.597216 | Mm.3879 | 12778166 |
| ID1 | transcription regulator | Hs.504609 | Mm.444 | 10537105 |
| ID3 | transcription regulator | Hs.76884 | Mm.110 | 10537105 |
| JUN | transcription regulator | Hs.696684 | Mm.275071 | [25] |
| KLF4 | transcription regulator | Hs.376206 | Mm.4325 | 16904174 |
| MYC | transcription regulator | Hs.202453 | Mm.2444 | 16904174 |
| NFKB1 | transcription regulator | Hs.618430 | Mm.256765 | [25] |
| NFKB2 | transcription regulator | Hs.73090 | Mm.102365 | [25] |
| NOTCH1 | transcription regulator | Hs.495473 | Mm.290610 | [25] |
| NOTCH2 | transcription regulator | Hs.487360 | Mm.254017 | [25] |
| NOTCH3 | transcription regulator | Hs.8546 | Mm.439741 | [25] |
| NOTCH4 | transcription regulator | Hs.436100 | Mm.173813 | [25] |
| POU5F1 | transcription regulator | Hs.249184 | Mm.17031 | 16904174 |
| RBPJ | transcription regulator | Hs.479396 | Mm.209292 | [25] |
| REL | transcription regulator | Hs.631886 | Mm.4869 | [25] |
| RELA | transcription regulator | Hs.502875 | Mm.249966 | [25] |
| RELB | transcription regulator | Hs.654402 | Mm.1741 | [25] |
| SOX2 | transcription regulator | Hs.518438 | Mm.65396 | 16904174 |
| STAT1 | transcription regulator | Hs.743244 | Mm.277406 | [25] |
| STAT3 | transcription regulator | Hs.463059 | Mm.249934 | [25] |
| Symbol | Growth factors and receptors | Human UniGene ID | Mouse UniGene ID | Reference/PubMed ID |
| CXCR1 | G-protein coupled receptor | Hs.194778 | Mm.337035 | [47] |
| CXCR2 | G-protein coupled receptor | Hs.846 | Mm.234466 | [47] |
| CXCR4 | G-protein coupled receptor | Hs.593413 | Mm.1401 | [25] |
| S1PR1 | G-protein coupled receptor | Hs.154210 | Mm.982 | [25] |
| ANGPT1 | growth factor | Hs.369675 | Mm.309336 | [33] |
| ANGPT2 | growth factor | Hs.583870 | Mm.439874 | [33] |
| ANGPTL3 | growth factor | Hs.209153 | Mm.28341 | 11877390 |
| CTGF | growth factor | Hs.410037 | Mm.390287 | [25] |
| EGF | growth factor | Hs.419815 | Mm.252481 | [47] |
| EREG | growth factor | Hs.115263 | Mm.4791 | 21794125 |
| FGF1 | growth factor | Hs.483635 | Mm.241282 | [25] |
| FGF2 | growth factor | Hs.284244 | Mm.473689 | [25] |
| FGF7 | growth factor | Hs.567268 | Mm.330557 | [25] |
| FIGF | growth factor | Hs.11392 | Mm.297978 | [25] |
| HGF | growth factor | Hs.396530 | Mm.267078 | [25] |
| IGF1 | growth factor | Hs.160562 | Mm.268521 | [25] |
| JAG1 | growth factor | Hs.626544 | Mm.22398 | [25] |
| JAG2 | growth factor | Hs.433445 | Mm.186146 | [25] |
| LEP | growth factor | Hs.194236 | Mm.277072 | 11460888 |
| MDK | growth factor | Hs.82045 | Mm.906 | 22707563 |
| PDGFA | growth factor | Hs.535898 | Mm.2675 | [25] |
| PDGFB | growth factor | Hs.1976 | Mm.144089 | [47] |
| PDGFC | growth factor | Hs.570855 | Mm.331089 | [47] |
| PGF | growth factor | Hs.252820 | Mm.4809 | [25] |
| TGFA | growth factor | Hs.170009 | Mm.137222 | [25] |
| TGFB1 | growth factor | Hs.645227 | Mm.248380 | [25] |
| TGFB2 | growth factor | Hs.133379 | Mm.18213 | [25] |
| VEGFA | growth factor | Hs.73793 | Mm.282184 | [33] |
| VEGFB | growth factor | Hs.732095 | Mm.15607 | [33] |
| VEGFC | growth factor | Hs.435215 | Mm.1402 | [33] |
| ITGAV | ion channel | Hs.436873 | Mm.227 | 18001496 |
| AKT1 | kinase | Hs.525622 | Mm.6645 | [25] |
| EFNA3 | kinase | Hs.516656 | Mm.331159 | 24180698 |
| EPHB4 | kinase | Hs.437008 | Mm.34533 | [47] |
| ERBB2 | kinase | Hs.446352 | Mm.290822 | 16489002 |
| FGFR3 | kinase | Hs.1420 | Mm.6904 | [25] |
| FLT1 | kinase | Hs.594454 | Mm.389712 | [33] |
| JAK2 | kinase | Hs.656213 | Mm.275839 | [25] |
| KDR | kinase | Hs.479756 | Mm.285 | [33] |
| MAP4K4 | kinase | Hs.701013 | Mm.19073 | [25] |
| MAPK1 | kinase | Hs.431850 | Mm.196581 | [25] |
| MAPK14 | kinase | Hs.485233 | Mm.311337 | [25] |
| MAPK8 | kinase | Hs.138211 | Mm.21495 | [25] |
| NRP2 | kinase | Hs.471200 | Mm.266341 | [33] |
| PIK3CA | kinase | Hs.553498 | Mm.260521 | [25] |
| PIK3CB | kinase | Hs.239818 | Mm.213128 | [25] |
| PIK3CD | kinase | Hs.518451 | Mm.229108 | [25] |
| PIK3CG | kinase | Hs.32942 | Mm.101369 | [25] |
| PTK2 | kinase | Hs.395482 | Mm.254494 | [25] |
| SPHK1 | kinase | Hs.68061 | Mm.20944 | [25] |
| TEK | kinase | Hs.89640 | Mm.14313 | [33] |
| TGFBR1 | kinase | Hs.494622 | Mm.197552 | [25] |
| TIE1 | kinase | Hs.78824 | Mm.4345 | [33] |
| ENG | transmembrane receptor | Hs.76753 | Mm.225297 | 21737653 |
| F3 | transmembrane receptor | Hs.62192 | Mm.273188 | 17898544 |
| FLT4 | transmembrane receptor | Hs.646917 | Mm.3291 | [33] |
| ICAM1 | transmembrane receptor | Hs.643447 | Mm.435508 | [25] |
| IGF1R | transmembrane receptor | Hs.643120 | Mm.275742 | [25] |
| ITGB3 | transmembrane receptor | Hs.218040 | Mm.87150 | 18579532 |
| KIT | transmembrane receptor | Hs.479754 | Mm.247073 | 21057534 |
| NRP1 | transmembrane receptor | Hs.131704 | Mm.271745 | [33] |
| VCAM1 | transmembrane receptor | Hs.109225 | Mm.76649 | [25] |
| ABCA1 | transporter | Hs.659274 | Mm.277376 | 23719382 |
| STAB1 | transporter | Hs.301989 | Mm.220821 | 22136669 |
| Symbol | Cytokines and chemokines | Human UniGene ID | Mouse UniGene ID | Reference/PubMed ID |
| CCL11 | cytokine | Hs.54460 | Mm.4686 | [25] |
| CCL2 | cytokine | Hs.303649 | Mm.290320 | [47] |
| CCL28 | cytokine | Hs.656904 | Mm.143745 | [25] |
| CCL5 | cytokine | Hs.514821 | Mm.284248 | [47] |
| CSF1 | cytokine | Hs.173894 | Mm.795 | [47] |
| CSF3 | cytokine | Hs.2233 | Mm.1238 | [25] |
| CX3CL1 | cytokine | Hs.531668 | Mm.103711 | [25] |
| CXCL1 | cytokine | Hs.789 | Mm.21013 | [25] |
| CXCL10 | cytokine | Hs.632586 | Mm.877 | [25] |
| CXCL11 | cytokine | Hs.632592 | Mm.131723 | [25] |
| CXCL12 | cytokine | Hs.522891 | Mm.303231 | [25] |
| CXCL13 | cytokine | Hs.100431 | Mm.10116 | [25] |
| CXCL14 | cytokine | Hs.483444 | Mm.30211 | [25] |
| CXCL16 | cytokine | Hs.745037 | Mm.425692 | [25] |
| CXCL3 | cytokine | Hs.89690 | Mm.244289 | [25] |
| CXCL5 | cytokine | Hs.89714 | Mm.4660 | [25] |
| CXCL9 | cytokine | Hs.77367 | Mm.766 | [25] |
| EDN1 | cytokine | Hs.713645 | Mm.14543 | 22409294 |
| IFNG | cytokine | Hs.856 | Mm.240327 | [25] |
| IL10 | cytokine | Hs.193717 | Mm.874 | [25] |
| IL12A | cytokine | Hs.673 | Mm.103783 | [25] |
| IL15 | cytokine | Hs.168132 | Mm.4392 | [25] |
| IL18 | cytokine | Hs.83077 | Mm.1410 | [25] |
| IL1B | cytokine | Hs.126256 | Mm.222830 | [25] |
| IL6 | cytokine | Hs.654458 | Mm.1019 | [25] |
| PF4 | cytokine | Hs.81564 | Mm.332490 | [25] |
| TNF | cytokine | Hs.241570 | Mm.1293 | [25] |
| Symbol | Proteases, inhibitors and others | Human UniGene ID | Mouse UniGene ID | Reference/PubMed ID |
| APOA1BP | enzyme | Hs.528320 | Mm.205996 | 23719382 |
| CDC42 | enzyme | Hs.467637 | Mm.1022 | 25584797 |
| DLL1 | enzyme | Hs.379912 | Mm.4875 | [25] |
| EGLN1 | enzyme | Hs.444450 | Mm.140619 | 18500244 |
| EGLN2 | enzyme | Hs.515417 | Mm.29978 | 18500244 |
| EGLN3 | enzyme | Hs.135507 | Mm.133037 | 18500244 |
| FN1 | enzyme | Hs.203717 | Mm.193099 | 20489157 |
| HPSE | enzyme | Hs.44227 | Mm.265786 | 16867222 |
| NOS3 | enzyme | Hs.647092 | Mm.258415 | 9616228 |
| PTGS1 | enzyme | Hs.201978 | Mm.275434 | 10581086 |
| PTGS2 | enzyme | Hs.196384 | Mm.292547 | 17072976 |
| TRAF2 | enzyme | Hs.522506 | Mm.3399 | [25] |
| TRAF6 | enzyme | Hs.591983 | Mm.292729 | [25] |
| ANGPTL4 | other | Hs.9613 | Mm.196189 | 15870027 |
| CD34 | other | Hs.374990 | Mm.29798 | 22249946 |
| CDH5 | other | Hs.76206 | Mm.21767 | 21528670 |
| CISH | other | Hs.655334 | Mm.4592 | [25] |
| COL18A1 | other | Hs.517356 | Mm.4352 | 17616861 |
| COL4A3 | other | Hs.570065 | Mm.389135 | 21528670 |
| DLL3 | other | Hs.127792 | Mm.12896 | [25] |
| DLL4 | other | Hs.511076 | Mm.143719 | [25] |
| DNAJA4 | other | Hs.513053 | Mm.28437 | 23142051 |
| EFNA1 | other | Hs.516664 | Mm.15675 | 17972146 |
| EFNB2 | other | Hs.149239 | Mm.209813 | [47] |
| IGFBP2 | other | Hs.438102 | Mm.141936 | 23233738 |
| LAMA5 | other | Hs.473256 | Mm.4339 | 24188612 |
| LECT1 | other | Hs.421391 | Mm.46561 | 14528519 |
| PECAM1 | other | Hs.514412 | Mm.343951 | 9616228 |
| PLXDC1 | other | Hs.125036 | Mm.39617 | 17031559 |
| PROK2 | other | Hs.528665 | Mm.87365 | 26317645 |
| SERPINE1 | other | Hs.414795 | Mm.250422 | 15695614 |
| SERPINF1 | other | Hs.532768 | Mm.2044 | 23486238 |
| THBS1 | other | Hs.164226 | Mm.4159 | 21528670 |
| THBS2 | other | Hs.371147 | Mm.26688 | 22553494 |
| TIMP1 | other | Hs.522632 | Mm.8245 | 24188612 |
| TIMP2 | other | Hs.633514 | Mm.206505 | 9462715 |
| TIMP3 | other | Hs.644633 | Mm.4871 | 12652295 |
| TNFAIP2 | other | Hs.525607 | Mm.255332 | [25] |
| ANPEP | peptidase | Hs.1239 | Mm.4487 | 17616861 |
| MMP2 | peptidase | Hs.513617 | Mm.29564 | 10225966 |
| MMP9 | peptidase | Hs.297413 | Mm.4406 | 10225966 |
| MMP14 | peptidase | Hs.2399 | Mm.280175 | 19208838 |
| PLAU | peptidase | Hs.77274 | Mm.4183 | 21528670 |
| PLG | peptidase | Hs.143436 | Mm.971 | 12911578 |
| SHH | peptidase | Hs.164537 | Mm.57202 | [47] |
| PTEN | phosphatase | Hs.500466 | Mm.245395 | [25] |
